# Preliminary Efficacy of Group Medical Nutrition Therapy and Motivational Interviewing among Obese African American Women with Type 2 Diabetes: A Pilot Study

**DOI:** 10.1155/2014/345941

**Published:** 2014-08-28

**Authors:** Stephania T. Miller, Veronica J. Oates, Malinda A. Brooks, Ayumi Shintani, Tebeb Gebretsadik, Darlene M. Jenkins

**Affiliations:** ^1^Department of Surgery, Meharry Medical College, 1005 Dr. D. B. Todd, Nashville, TN 37208, USA; ^2^Department of Family and Consumer Sciences, College of Agriculture, Human and Natural Sciences, Tennessee State University, 224 Humphries Hall, 3500 John A. Merritt Boulevard, Nashville, TN 37209, USA; ^3^Department of Biostatistics, Vanderbilt University Medical Center, S-2323 Medical Center North, Nashville, TN 37232-2158, USA; ^4^National Health Care for the Homeless Council, P.O. Box 60427, Nashville, TN 37206, USA

## Abstract

*Objective*. To assess the efficacy and acceptability of a group medical nutritional therapy (MNT) intervention, using motivational interviewing (MI). *Research Design & Method*. African American (AA) women with type 2 diabetes (T2D) participated in five, certified diabetes educator/dietitian-facilitated intervention sessions targeting carbohydrate, fat, and fruit/vegetable intake and management. Motivation-based activities centered on exploration of dietary ambivalence and the relationships between diet and personal strengths. Repeated pre- and post-intervention, psychosocial, dietary self-care, and clinical outcomes were collected and analyzed using generalized least squares regression. An acceptability assessment was administered after intervention. *Results*. Participants (*n* = 24) were mostly of middle age (mean age 50.8 ± 6.3) with an average BMI of 39 ± 6.5. Compared to a gradual pre-intervention loss of HbA1c control and confidence in choosing restaurant foods, a significant post-intervention improvement in HbA1c (*P* = 0.03) and a near significant (*P* = 0.06) increase in confidence in choosing restaurant foods were observed with both returning to pre-intervention levels. 100% reported that they would recommend the study to other AA women with type 2 diabetes. *Conclusion*. The results support the potential efficacy of a group MNT/MI intervention in improving glycemic control and dietary self-care-related confidence in overweight/obese AA women with type 2 diabetes.

## 1. Introduction

Medical nutrition therapy (MNT) is an established and effective method for improving dietary intake and clinical outcomes in patients with diabetes [1]. The main goals are to support patients in achieving and maintaining glycemic, blood pressure, and lipid control. An additional goal is to give attention to patients' personal preferences, culture, and willingness to make dietary changes and their ability to maintain the pleasure of eating [2]. It is this latter goal that is particularly important among patients for whom dietary intake is intimately associated with cultural patterns and traditions [3, 4], such as African Americans.

There is little evidence that MNT interventions targeting African American (AA) women with diabetes, a group at high risk for development and progression of diabetes-related complications [5], have addressed motivation for engaging in healthier dietary intake patterns. Enhancing motivation for dietary-related behaviors, such as better selection and preparation of food choices, is important given the reported challenges and barriers to achieving optimal health among this subpopulation. Identified challenges and barriers include intake of a “soul food diet” which, in many instances, includes foods that are high in saturated fats and simple sugars [6]; cultural orientation [7]; and multicaregiver roles [8]. Here, we describe the design and results of a pilot study to assess the efficacy and acceptability of a group MNT intervention, using motivational interviewing strategies [9], among AA women with type 2 diabetes.

## 2. Methods

### 2.1. Design

We used a one-arm interrupted time series design, a quasiexperimental research design [10–12] in which a series of pre-intervention assessments were conducted (18, 12, and 6 weeks prior to intervention and baseline), followed by a group, diabetes educator/dietitian-facilitated intervention and a series of post-intervention assessments (6, 12, and 18 weeks after intervention) (Box 1). Pre- and post-intervention assessment visits were conducted at Meharry Medical College based on participants' schedules and intervention study sessions were conducted in the evening hours at a collaborating YMCA facility. To offset any participation-related expenses, cash was provided for assessment visits and intervention sessions. The Meharry Medical College Institutional Review Board approved all study informed consent and intervention procedures.

### 2.2. Participants

African American women meeting the following criteria were recruited via referral from a managed care organization and using radio, newspaper, and email advertisements: (1) 34-year-old-and-older age group most likely to have numerous competing priorities (i.e., multicaregiver roles) that may make dietary self-care difficult; (2) type 2 diabetes diagnosis for 6 months or longer; and (3) at risk for microvascular and macrovascular diabetes-related complications (HbA1c ≥ 7.0%) and either current systolic blood pressure of ≥130, LDL cholesterol ≥ 100, or BMI ≥ 30. The research assistant prescreened, via telephone, women responding to all recruitment methods and scheduled in-person visits to assess inclusion criteria, including HbA1c.

### 2.3. Interventionist Training and Intervention Implementation

The PI consulted with a professional MI trainer to develop group MI activities that were consistent with MNT goals. The diabetes educator and dietitians participated in a full day, group, face-to-face MI training session followed by 2 telephone training calls. Training covered basic MI principles and strategies and role-playing exercises were used for skill reinforcement and protocol-guided practice.

The group MNT, MI intervention consisted of 5 sessions (Table 1) which included a didactic education component, a hands-on skills building component with homework assignments, and an MI component. The goal of session 1 was to enhance participants' motivation for the subsequent sessions by exploring their study expectations (dietitian-facilitated) and to provide an overview of the 7 key diabetes self-care behaviors as recommended by the American Association of Diabetes Educators [13] (diabetes educator-facilitated). The activities for the next 3 sessions centered on managing carbohydrate, fruit and vegetable, and dietary fat intake. The session goals were to provide education, demonstrate and support self-care strategies, and cultivate motivation for and increase self-efficacy in utilizing management strategies. As an example of the MI component, participants ranked their perception of the importance of managing dietary intake and elaborated on the rationale for their responses (session 2). This activity was based on the premise that motivational readiness is likely to be greater when the behavior change is important to the individual [14]. Other motivational strategies included exploration and discussion of dietary ambivalence and linking personal strengths to dietary self-care. The goal of the 5th session was to aid participants in developing a plan for long-term dietary self-care based on strategies learned and their own personal successes throughout the intervention.

### 2.4. Measures

With the exception of demographic data, diabetes medical history, and dietary intake patterns/concerns, all measures were administered at baseline and 18, 12, and 6 weeks before and after intervention.

*Baseline Demographic, Diabetes Medical History, and Dietary Intake Patterns/Concerns*. A 7-item questionnaire was used to assess basic demographic information and a 12-item questionnaire was used to assess diabetes medical history. One question was used to assess major dietary concerns (e.g., eating late at night) and 5 questions assessed frequency of food restriction to lose weight, general overeating, eating unplanned snacks, emotional eating, and eating out. The latter five questions were used as part of the Southern Community Cohort Study [16], an ongoing cohort study examining cancer and other chronic disease racial disparities among lower-income African Americans and Caucasians.

*Self-Care and Psychosocial Measures. *Questions from theexpanded Summary of Diabetes Self-Care Behaviors [17] questionnaire were used to assess number of days/week participants engaged in specific dietary behaviors targeted during the intervention (e.g., carbohydrates spacing throughout the day and fruit and vegetable and fat intake) and general eating behaviors. Confidence in making food choices while dining out was measured using a question from the Confidence in Diabetes questionnaire [18]. Test-retest reliability, using pre-intervention time point assessment results, was acceptable for all questions. For dietary self-care, Pearson correlations ranged from 0.56 to 0.75, consistent with reliability estimates ranging from 0.42 to 0.67 in previous studies [17]. Pearson correlations ranged from 0.520 to 0.77 for confidence in dining out.

*Physiologic Measures. *Venous, fasting blood draws were used for HbA1c laboratory assessments.* Blood pressure* was measured with a calibrated sphygmometer after participants had been at rest for 15 minutes. BMI was calculated from weight and height measurements.

*Acceptability. *At the conclusion of the final intervention session, participants completed an 18-item written evaluation which assessed helpfulness, strengths, and weaknesses of intervention components and previous exposure to information shared during the didactic education component. The evaluation included both closed-ended Likert-format questions and open-ended questions.

### 2.5. Statistical Analysis

Descriptive statistics are presented as either means and standard deviations or medians and interquartile ranges for continuous variables and proportions for categorical variables. Intervention efficacy was assessed by comparing rate and direction of change (slope) in outcome variables as quantified by the regression coefficient between pre-intervention (baseline and 18, 12, and 6 weeks before intervention) and post-intervention phase (18, 12, and 6 weeks after intervention). Generalized least squares regression (GLS) modeling was used to account for the correlated data (repeated measures). In the GLS model, to allow the detection of different trends when comparing the pre- and post-intervention periods outcomes, an interrupted time-series analysis was [19] used in which we included a cross-product term of a period indicator for pre- and post-intervention with time of follow-up (test of interaction).

## 3. Results and Discussion

### 3.1. Participants Characteristics

In collaboration with the MCO and via radio ads, a newspaper ad, and email blasts, 24 participants accepted enrollment invitations (71% from MCO) (Table 2). We recruited a primarily middle age (50.8 ± 6.3 years) group with up to 50% reporting some type of diabetes-related complication including delayed gastric emptying (18.2%), vision problems (26.1%), and neuropathy (50%). The most common, significant dietary intake concerns were unhealthy food cravings (36.4%), eating too much (31.8%), and eating late at night (31.8). The most frequent (at least 1 time per day) dietary intake pattern was food restriction to lose weight (30.4%). Baseline BMI and HbA1c measures, by design based on inclusion criteria (Table 3; −18 weeks), reflect an obese participant group with average HbA1c levels above the recommended 7%.

### 3.2. Intervention Efficacy

Table 3 shows the results of self-care, psychosocial, and physiologic assessments at study entry, 18 weeks prior to the intervention (−18 weeks) and throughout the study. Compared to a progressive loss of HbA1c control (8.8 ± 2.0 to 9.7 ± 2.4) between pre-intervention time points, there was a statistically significant (*P* = 0.029) improvement between post-intervention time points with levels returning to baseline (9.4 ± 1.8 to 8.8 ± 2.2). There were no significant changes in other physiologic outcome variables (e.g., BMI). For confidence in choosing foods while dining out, compared to decreasing pre-intervention confidence levels (1.9 ± 0.9 to 2.05 ± 0.8), there was a near significant (*P* = 0.055) increase between post-intervention time points that returned to baseline levels (2.1 ± 0.9 to 1.8 ± 0.6). For number of days/week eating five fruits and vegetables, compared to increasing pre-intervention consumption levels (3.9 ± 1.8 to 4.5 ± 1.7 days/week), significantly different (*P* = 0.016) decreasing levels of consumption were observed after intervention (4.8 ± 1.8 to 4.0 ± 1.9 days/week). For number of days engaging in a generally healthy diet, pre-intervention engagement increased over time (3.3 ± 1.6 to 4.4 ± 1.6 days/week) compared to a statistically significant (*P* = 0.035) decrease in engagement during post-intervention time points (5.0 ± 1.8 to 4.3 ± 1.9 days/week). Though there were no significant differences for days/week spacing carbohydrates throughout the day and eating high fat foods, post-intervention engagement was improved relative to pre-intervention engagement for both behaviors. For example, for carbohydrate spacing throughout the day, the highest pre-intervention level of engagement (3.5 ± 1.8 days/week) was less than the lowest level of post-intervention engagement (3.9 ± 1.9 days/week) which extended up to 4.3 ± 2.2 days/week.

### 3.3. Intervention Acceptability

Greater than 95% of the participants viewed each component of the MNT-related information (e.g., fruits/vegetables, fats) as mostly helpful or very helpful and 100% reported that they would recommend the intervention to other women. One participant voiced her willingness to recommend the intervention by stating, “…I tried to recruit for this study.” Learning how carbohydrate impacts blood sugar and the difference between fat types were commonly reported as the most helpful informational components. Ninety-four percent of participants reported that the carbohydrate-related information was completely or mostly new to them compared to 81% and 56% for dietary fat and fruit and vegetable intake, respectively. Absence of grocery shopping lists and more meal ideas were among perceived shortcomings. The following written comments reflect common participant sentiments: “…The discussions were pivotal in my process. Sometimes, I've felt alone in all this.”; “I enjoyed meeting other women of my ethnic background that have diabetes that struggle to control it.”

### 3.4. Discussion

These pilot data indicate that a group MNT/MI intervention may improve glycemic control among AA women with type 2 diabetes who are at high risk of the development or progression of diabetes-related complications. The importance of glycemic control in preventing or reducing the severity of complications is well documented [20]. It is also widely known that AAs are among subgroups at highest risk of complications due to poor glycemic control [21, 22]. While our findings of improved glycemic control require evaluation in a larger sample with a longer follow-up, they do support the feasibility and potential efficacy among high risk subgroups.

The post-intervention trend in improved confidence in choosing foods while dining out provides some evidence of intervention efficacy in improving a psychosocial outcome that may precede dietary change or clinical outcomes. This hypothesis is supported by our previous work in AA women with type 2 diabetes showing a statistically significant correlation between lack of confidence in diabetes self-care behaviors and reduced glycemic control [23].

The post-intervention decreases in number of days eating an overall healthy diet and 5 servings of fruits and vegetables were not expected. It is possible that additional information learned during the didactic intervention components caused participants to be more aware of their self-care behaviors that were not consistent with current recommendations. For example, learning about what constitutes a healthy diet might result in participants self-reporting less engagement as they endeavor to apply the new knowledge to daily dietary intake. Since the fruit and vegetable intake question asked about both food types, it is not possible to delineate which food type participants were referring to in their responses. However, it is a line of inquiry that is deserving of further exploration (e.g., use of a measure to assess fruit and vegetable intake separately) particularly given the variation in carbohydrate content in different fruits and vegetables [24].

Though the observations that carbohydrate spacing throughout the day and limiting dietary fat improved did not show a statistically significant shift, they are behaviorally relevant such that post-intervention engagement in these specific behaviors was improved compared to pre-intervention engagement. In essence, these results indicate that the intervention promoted greater engagement in a targeted intervention behavior for which participants were already incorporating into daily life.

In addition to a high degree of acceptability, it is noteworthy that the majority of the participants reported the information shared during the didactic educational components as new knowledge. This is particularly salient for carbohydrate-related information, which the greatest percentage of participants reported as new information, given that this food category has significant impact on glucose control [1]. Though we did not assess which components of carbohydrate education were new, frequent comments about the helpfulness of learning about the physiological relationship between carbohydrate intake and glycemic control may have influenced responses.

This study has several strengths. No studies, to our knowledge, have reported the application of a group (versus individual) MI intervention approach among AA women with type 2 diabetes exclusively. From a translational standpoint and given the evidence of positive impact on glycemic control, group MI interventions may be more cost-effective, particularly in settings where there are insufficient financial and personnel resources to support individual consultations. Our interrupted time-series analysis allowed for observations of post-intervention trends, such as gradual improvement in glycemic control that would not have been possible using a single pre- and post-intervention design. This is particularly relevant for lifestyle interventions in groups at higher risk for development or progression of diabetes complications as these individuals may have more barriers to self-care engagement than those with lower risk profiles. An additional strength was the successful recruitment of a predominantly lower socioeconomic status participant group given previously described participation barriers (e.g., transportation and childcare) [25].

This study was not without limitations. There was not a comparison group. However, our use of the interrupted time-series design, a quasiexperimental research design, is highly endorsed for behavioral and translational interventions [10–12] for which there is insufficient evidence to warrant application to a larger sample. Given the initial evidence of efficacy described here, future effectiveness studies should include a comparison intervention. Second, because of the various recruitment methods, we did not recruit a homogenous sample of women relative to socioeconomic status, a factor which is reported to have substantial impact on diabetes lifestyle behaviors [26, 27]. Therefore, our study is generalizable to AA women from various socioeconomic backgrounds with type 2 diabetes and who are at risk for the development or progression of diabetes-related complications. Though the sample size was small, it was consistent with our goals to establish preliminary efficacy of the intervention and yielded important and unexpected findings that will aid in implementing and optimizing a subsequent effectiveness study.

## 4. Conclusion

We have described the design and results of a study to assess the efficacy and acceptability of translating a group MNT/MI intervention among AA women with type 2 diabetes. Our findings warrant a full-scale randomized clinical trial to evaluate the effectiveness of the intervention to promote improvements in diabetes psychosocial, self-care, and clinical outcomes and to evaluate the relative influence of different intervention components on outcomes of interest.

## Figures and Tables

**Box 1:**
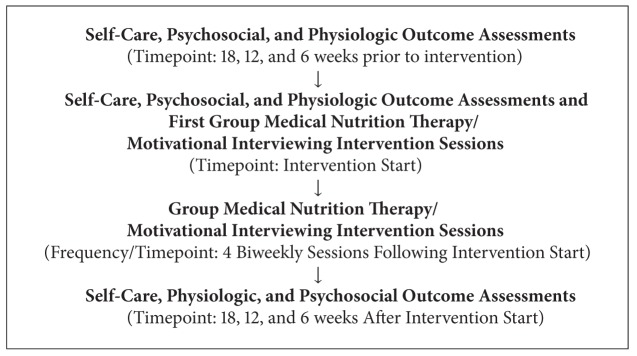
Research Design Overview. Group medical nutrition therapy/motivational interviewing intervention among African American women with type 2 diabetes.

**Table 1:** Group medical nutrition therapy/motivational interviewing intervention among African American women with type 2 diabetes.

Motivational interviewing component	Medical nutritional therapy education/behavioral skills component
	Session 1
Dietitian-led discussion of participants study expectations (served as ice-breaker)	(i) Certified diabetes educator-led diabetes self-management overview (ii) Homework presentation/discussion (record a single breakfast, lunch, and dinner meal)

	Session 2
Dietitian-led “importance” exercise—participants ranked and discussed their perceptions of the importance of managing dietary intake	(i) Dietitian-led carbohydrate management presentation (carbohydrate/blood glucose relationship, identification of carbohydrate food sources; basic carbohydrate counting) (ii) Meal planning using “plate” method and food models (ii) Homework presentation/discussion (drink 64 ounces of water; record all meals for 3 days/week including a weekend day)

	Session 3
Dietitian-led “roadmap” exercise—participants used a picture of a roadmap to discuss pros and cons of 2 paths: (1) limiting dietary fat; (2) not limiting dietary fat intake	(i) Dietitian-led dietary fat presentation (fat/heart health relationship, identification of different fat types with emphasis on reducing saturated/trans fats, identification of fat content of popular restaurant foods) (ii) Use of participant- and dietitian-provided food labels and food models to identify fat/types and quantities intake (iii) Homework presentation/discussion (same as session 2 above in addition to recording carbohydrate and fat content of meals)

	Session 4
Dietitian-led “strength” exercise—participants selected and described their personal strengths and elaborated on how strengths could be used in managing dietary intake	(i) Dietitian-led carbohydrate management presentation (carbohydrate/blood glucose relationship, identification of carbohydrate food sources; basic carbohydrate counting) (ii) Meal planning using “plate” method and food models (iii) Homework presentation/discussion (same as session 3 in addition to planning 3 meals in advance and recording types of fat; identify dietary management strategies that have been most helpful to date for use in long-term planning)

	Session 5
Dietitian-led discussion of participants' dietary successes/improvements since the beginning of the intervention	(i) Dietitian-led long-term planning exercise (identify dietary goal based on strategies that have been most helpful; identification of specific activities to accomplish goals; development of an action plan) (ii) Action plan based on strategies from “Living a Healthy Life with Chronic Conditions” [15]

**Table 2:** Baseline participant characteristics (*N* = 24).

Demographic and clinical	
Age—years (mean, SD)	50.8 (6.3)
Income (%)	
<10,000	34.8
10,000–20,000	17.4
20,000–40,000	8.7
40,000–60,000	13.0
>60,000	8.7
No answer	17.4
Employment (%)	
Work outside the home full-time	43.4
Work outside the home part-time	8.6
No job outside the home	34.7
Retired	4.3
Other (disabled; home business/self-employed)	30.4
Marital status—single (%)	37.5
Diabetes duration—years (median, IQR)	6 (3.75, 12)
Vision problems (%)	26.1
Numbness in feet or legs (%)	50
Delayed gastric emptying (%)	18.2

Dietary intake concerns	
Reported the following as “most significant dietary issue” (%)	
Frequent cravings for unhealthy food or snacks	36.4
Not eating enough fruit	13.6
Eating too much	31.8
Not eating enough vegetables	4.5
Eating fried foods or other foods high in fat	22.7
Eating late at night	31.8
Other (not really knowing what to eat)	4.5

Usual eating habits	
Reported the following at least 1 time per day (%)	
Food restriction to lose weight	30.4
Overeat	8.6
Eat unplanned snacks	8.7
Eat to cope with negative feelings	8.7
Eat at restaurants (including fast food)	4.3

**Table 3:** Group medical nutrition therapy/motivational interviewing intervention among African American women with type 2 diabetes: impact on self-care, psychosocial, and physiologic outcomes.

Outcome variable	pre-intervention values				post-intervention values			*P* value∗
	−18 weeks	−12 weeks	−6 weeks	0	6 weeks	12 weeks	18 weeks	
Carbohydrate spacing (days/week)∗∗	2.0 ± 1.9	3.5 ± 1.8	2.9 ± 1.8	3.4 ± 2.0	3.9 ± 1.9	4.3 ± 2.2	4.1 ± 2.0	0.277
Eat 5 fruit/vegetables (days/week)∗∗	3.9 ± 1.8	4.2 ± 1.6	3.9 ± 1.8	4.5 ± 1.7	4.8 ± 1.8	4.7 ± 1.6	4.0 ± 1.9	0.016
Eat high fat food (days/week)	3.8 ± 1.6	3.5 ± 1.8	3.4 ± 1.7	3.7 ± 1.9	3.0 ± 1.9	2.4 ± 1.6	2.9 ± 1.9	0.992
Eat a generally healthy diet (days/week)∗∗	3.3 ± 1.6	4.9 ± 1.3	4.4 ± 1.5	4.4 ± 1.6	5.0 ± 1.5	4.8 ± 1.7	4.3 ± 1.9	0.035
Confidence in choosing restaurant foods (score)∗∗∗	1.9 ± 0.9	2.0 ± 0.9	2.2 ± 0.9	2.05 ± 0.8	2.1 ± 0.9	1.8 ± 0.9	1.8 ± 0.6	0.055
HbA1c (%)	8.8 ± 2.0	8.3 ± 1.9	9.2 ± 2.1	9.7 ± 2.4	9.4 ± 1.8	9.3 ± 1.8	8.8 ± 2.2	0.029
BMI kg/m^2^	39 ± 6.5	39.7 ± 6.9	39.6 ± 6.5	39.4 ± 6.8	38.6 ± 6.5	39.2 ± 6.5	38.9 ± 6.8	0.407
Systolic blood pressure (mm/Hg)	139 ± 19	134 ± 19	139 ± 15	143 ± 16	141 ± 22	139 ± 17	141 ± 26	0.384
Diastolic blood pressure (mm/Hg)	84.1 ± 7.8	82.2 ± 8.2	86.0 ± 10.9	84.6 ± 10.8	84.0 ± 10.5	80.9 ± 7.0	81.0 ± 9.7	0.091

*Generalized least squares regression was used to compare rate of change in outcomes quantified as the regression coefficient between pre- and post-intervention phases and to account for repeated measures. ∗∗From Summary of Diabetes Self-Care activities question; score range 0 to 7 days/week. ∗∗∗From Confidence in Diabetes Questionnaire [18]; score range from 1 (very confident) to 5 (not confident).
